# Coming of Age for BTK Inhibitor Therapy: A Review of Zanubrutinib in Waldenström Macroglobulinemia

**DOI:** 10.3390/cells11203287

**Published:** 2022-10-19

**Authors:** Javier Muñoz, Jonas Paludo, Shayna Sarosiek, Jorge J. Castillo

**Affiliations:** 1 Department of Hematology, Mayo Clinic, Phoenix, AZ 85054, USA; 2 Department of Hematology, Mayo Clinic, Rochester, MN 55905, USA; 3 Bing Center for Waldenström’s Macroglobulinemia, Dana-Farber Cancer Institute, Harvard Medical School, Boston, MA 02215, USA

**Keywords:** Waldenström macroglobulinemia, zanubrutinib, Bruton tyrosine kinase inhibitor, costs, combination therapy

## Abstract

Waldenström macroglobulinemia (WM) is a rare form of non-Hodgkin B-cell lymphoma with a variable clinical presentation that can impact a patient’s quality of life by causing anemia, peripheral neuropathy, serum hyperviscosity, extramedullary disease, and other symptoms. There are several safe and effective treatment regimens for patients with WM, and the choice of therapy should be made in a personalized fashion considering the patient’s symptoms, comorbidities, and genomic profile. Bruton tyrosine kinase (BTK) inhibitors are a new option to treat patients with WM. Zanubrutinib is a next-generation covalent BTK inhibitor designed to have fewer off-target effects than previous BTK inhibitors. This review summarizes the pharmacokinetic and pharmacodynamic properties of zanubrutinib as well as safety and efficacy findings. Then, it explores the health economic and outcomes research associated with the costs of treating patients with WM and the reasons why zanubrutinib may be a more cost-effective treatment option compared with ibrutinib, a first-generation BTK inhibitor. Future directions for the treatment of WM focus on the use of zanubrutinib in combination therapy. Combinations based on effective ibrutinib or acalabrutinib treatments may be effectively applied with zanubrutinib given the similar mechanism of action for these BTK inhibitors. Combination therapies could also help prevent the development of disease resistance, minimize toxicity, and support treatment regimens of finite duration.

## 1. Introduction: Background on Waldenström Macroglobulinemia

Waldenström macroglobulinemia (WM) is a rare, incurable, non-Hodgkin B-cell lymphoma caused by the malignant accumulation of lymphoplasmacytic lymphoma cells in bone marrow and other organs, which secrete a monoclonal immunoglobulin M paraprotein [1,2]. In the US and Europe, the incidence of WM is 3 to 4 cases annually per million people [2,3,4]. It is more common in White men over 60 years of age [2,3,5]. Common symptoms of WM include serum hyperviscosity, B symptoms, bleeding, and anemia; however, approximately a quarter of patients are asymptomatic at the time of diagnosis [4,6]. The anemia associated with WM is attributed to insufficient erythropoiesis due to bone marrow infiltration and low iron levels [6,7]. Additionally, precipitation of immunoglobulin M can cause peripheral neuropathy, cryoglobulinemia, and cold agglutinin syndrome [6]. Rare features of WM include malignant pleural effusions [8], renal disease [9], and central nervous system involvement [10].

Recurring somatic mutations have been observed in patients with WM [11]. The *MYD88* innate immune signal transduction adaptor (*MYD88*) L265P mutation, detected in approximately 90% of patients with WM [12], mediates the activation of nuclear factor κB via interleukin-1 receptor-associated kinase 1 and Bruton tyrosine kinase (BTK), which promotes the survival of WM cells [13]. C-X-C motif chemokine receptor 4 (*CXCR4*) mutations are also present in up to 40% of patients with WM [14]; mutations in *CXCR4* promote sustained activation of the 2 kinase signaling pathways involved in survival (serine/threonine-protein kinase Akt and extracellular signal-regulated kinase). *CXCR4* mutations occur subclonally in patients who have *MYD88* mutations. The presence of *CXCR4* mutations has been associated with higher serum immunoglobulin M levels and an increased risk of hyperviscosity [15].

Selection of a treatment regimen for WM includes a review of treatment efficacy and safety, but patient-specific factors, such as a patient’s mutational profile, comorbidities, and preference must also be considered [16]. Rituximab-based treatments are common for WM, particularly for treatment-naive (TN) disease. Some patients with WM will eventually experience disease progression and need a new treatment regimen or may be poor candidates for certain treatment regimens. This is particularly relevant in light of data showing that patients with B-cell malignancies, such as chronic lymphocytic leukemia (CLL), are at a high risk of death if they require inpatient admission for symptomatic coronavirus disease 2019 (COVID-19) [17], and they have a poor response to COVID-19 vaccination [18]. For these patients, it may be better to avoid treatment regimens that are administered in a clinic or hospital setting where exposure to the disease could occur.

Covalent BTK inhibitors are a newer option for the treatment of WM [1]. BTK is involved in the signaling cascade for B-cell malignancies downstream of the B-cell antigen receptor and is essential for the development and function of B cells [19]. Inhibition of BTK has shown to induce apoptosis in WM cells, and when inhibited in combination with interleukin-1 receptor-associated kinase 1 and 4, improved inhibition of nuclear factor κB signaling occurred along with WM cell death [13].

Three orally administered covalent BTK inhibitors are approved by the US Food and Drug Administration (FDA) as treatment options for B-cell malignancies: ibrutinib, acalabrutinib, and zanubrutinib [20,21,22]. Ibrutinib is a first-generation BTK inhibitor administered once daily and was the first BTK inhibitor to receive FDA approval for the treatment of WM [22]. Ibrutinib is also approved to treat mantle cell lymphoma (MCL) in patients who have had at least 1 prior treatment, CLL/small lymphocytic lymphoma (SLL), marginal zone lymphoma in patients who require systemic therapy and have had 1 prior anti-CD20-based therapy, and chronic graft versus host disease after failure of a systemic therapy. Ibrutinib induces high response rates in patients with WM, with an overall response rate (ORR) as high as 100% (*n* = 30) observed in patients with TN WM [23] and 90.5% (*n* = 63) in patients with relapsed/refractory (R/R) disease [24]. However, ibrutinib also has well-described off-target effects, including atrial fibrillation, hypertension, and hemorrhage [24,25], and evidence shows that some patients with WM require a dose reduction (~20%; *n* = 95 and 15) [26,27] or treatment discontinuation (31% [*n* = 25], 68% of which were due to treatment-related toxicities) [27]. The discontinuation rate of ibrutinib in patients with CLL in real-world settings has a wide range, from 16% (of 1497 patients) to 50% (of 447 patients) [28,29,30]. 

Acalabrutinib is a second-generation BTK inhibitor administered twice daily [20] and has fewer off-target effects compared with ibrutinib [31]. Acalabrutinib is FDA approved to treat MCL in patients who have received at least 1 prior therapy and CLL/SLL [20]; it also has a high ORR in patients with both TN (93% [95% CI, 66–100%]; *n* = 14) and R/R (93% [95% CI, 86–98%]; *n* = 92) WM [32]. Discontinuation remained a problem though, with 50% of patients with TN disease and 25% of patients with R/R disease having discontinued treatment due to any cause during the study. Adverse events led to discontinuation in 7% of patients (*n* = 106). Grade 3/4 atrial fibrillation occurred in only 1 patient (1%), and grade 3/4 bleeding occurred in 3 (3%). A direct comparison with other BTK inhibitors in WM has not yet been performed [32], although a randomized trial comparing acalabrutinib (*n* = 268) to ibrutinib (*n* = 265) in patients with R/R CLL showed that acalabrutinib had a better adverse effect profile than ibrutinib [33]. 

Zanubrutinib is a next-generation BTK inhibitor administered once or twice daily [21]. It was designed to have fewer off-target effects [34] and is approved by the FDA for the treatment of patients with MCL who have received prior therapy and patients with R/R marginal zone lymphoma who have had at least 1 anti-CD20–based treatment [21]. Zanubrutinib is also approved by the FDA for use in WM [35]. 

In 2020, a thorough review describing research on zanubrutinib to treat WM was published [36]. There have been many advances in WM treatment since then. Another review published in 2022 focused on updated safety and efficacy data from clinical trials [37]. Here, we briefly review background information on zanubrutinib use in patients with WM and developments since these recent publications, then we focus on additional research related to patient treatment, including data on health economics and outcomes research, and future directions for WM treatment.

## 2. Zanubrutinib Pharmacokinetic (PK) and Pharmacodynamic Data

In a phase 1 dose-escalation study of zanubrutinib in patients with B-cell malignancies, including WM, it was found that zanubrutinib was rapidly absorbed after oral administration, reaching maximum concentration in approximately 2 h [38]. The mean half-life of zanubrutinib was 4 h when dosed at 160 mg twice daily or 320 mg once daily, with minimal accumulation observed after repeated dosing. BTK occupancy > 95% was observed 4 h after dosing at levels as low as 40 mg per day, which indicated that occupancy was maintained after reaching peak plasma levels. More patients in the study achieved > 95% sustained BTK occupancy in lymph nodes when zanubrutinib was administered at 160 mg twice daily compared with 320 mg once daily; however, the clinical relevance of having high BTK inhibitor occupancy in lymph nodes is unknown. Both dosing regimens were well tolerated.

### Recent Developments

A cocktail probe approach was used to assess drug–drug interactions of cytochrome P450 (CYP) enzymes and drug transporter proteins on zanubrutinib PK (*n* = 18) [39]. At 320 mg total per day, zanubrutinib had little to no effect on warfarin, rosuvastatin, or digoxin, which are the PK probes of CYP2C9, breast cancer resistance protein, and P-glycoprotein, respectively. Zanubrutinib decreased systemic exposure of midazolam and omeprazole (CYP3A and CYP2C19 substrates, respectively).

A clinical trial to assess the use of CYP3A inducers (*n* = 20) or inhibitors (*n* = 18) in combination with zanubrutinib in Asian and non-Asian patients verified that zanubrutinib is primarily metabolized by CYP3A [40]. The study also showed that rifampin, a strong CYP3A inducer, affected bioavailability and clearance of zanubrutinib, causing a 13.5-fold decrease in AUC_0-∞_ and a 12.6-fold decrease in the maximum serum concentration; therefore, it was recommended that strong CYP3A inducers should not be administered with zanubrutinib due to the decrease in zanubrutinib exposure that could impact its efficacy. The strong CYP3A inhibitor itraconazole increased exposure to zanubrutinib 3.8-fold for AUC_0-∞_ and 2.6-fold for the maximum serum concentration. Since fungal and bacterial infections are common in patients with B-cell malignancies, a reduced zanubrutinib dosage was recommended when coadministered with treatments that are strong CYP3A inhibitors to avoid exceeding the exposure observed with the maximum clinically tested dose of 320 mg daily. The US prescribing information for acalabrutinib indicates avoidance of concomitant use with strong CYP3A inhibitors [20], while ibrutinib can only be used concomitantly with posaconazole and voriconazole [22]. The clinical trial also verified that no PK differences were observed between Asian and non-Asian patients who received zanubrutinib [40]. This was supported by a separate modeling study that showed no dosage modifications were needed based on race or other patient characteristics such as age, sex, body weight, mild or moderate renal impairment, tumor type, or use of acid-reducing agents [41].

## 3. Zanubrutinib Safety and Efficacy

A pooled safety analysis assessed treatment-emergent adverse events (TEAEs) and treatment-limiting toxicities of zanubrutinib in patients with B-cell malignancies (*n* = 779) [42]. Most patients (98%) experienced TEAEs, with 66% reporting at least 1 TEAE that was grade ≥ 3, including 37% with treatment-related events. Nonhematologic TEAEs (incidence ≥ 15%) included upper respiratory tract infection (39%), rash (27%), bruising (25%), musculoskeletal pain (24%), diarrhea (23%), cough (21%), pneumonia (21%), urinary tract infection (15%), and fatigue (15%). Hematologic AEs were reported as AEs of interest and included bleeding or bruising (55%), treatment-emergent neutropenia (36%), thrombocytopenia (21%), and anemia (18%). At least 1 serious AE was reported in 46% of patients, including 17% that were treatment related. Serious AEs included pneumonia (11%); cellulitis, sepsis, urinary tract infection, upper respiratory tract infection, and pyrexia (2% each); and febrile neutropenia (1%). One or more dose reductions occurred in 8% of patients; common TEAEs that led to a dose reduction were neutropenia, diarrhea, and pneumonia (1% each). The most common cause of treatment discontinuation was progressive disease (27%). TEAEs led to treatment discontinuation in 10% of patients; almost half of the TEAEs were treatment related, including pneumonia (2%) and hemorrhage (1%).

In patients with WM in the pooled safety analysis, the discontinuation rate due to TEAEs was 10%, and infections were common (80%), with 8 patients having 1 or more opportunistic infections, including 1 death [42]. Over half (54%) of the patients with WM reported bleeding events, and major hemorrhages occurred in 6% of patients. Neutropenia and thrombocytopenia occurred in 32% and 15% of patients with WM, respectively.

In a phase 1/2 study of zanubrutinib in patients with TN (*n* = 24) or R/R WM (*n* = 53), the ORR was 96% at follow-up (median, 36 and 23.5 months for patients with R/R and TN WM, respectively) with 73% of patients remaining on treatment [43]. The estimated 3-year progression-free survival (PFS) rate was 80.5%, with a 3-year overall survival (OS) rate of 85%. AEs led to treatment discontinuation in 13% of patients; 1 patient discontinued treatment due to a treatment-related AE. AEs of interest included contusion (32.5%), neutropenia (19%), major hemorrhage (4%), atrial fibrillation/flutter (5%), and grade 3 diarrhea (3%).

The phase 3 ASPEN trial compared the use of zanubrutinib with ibrutinib in patients with WM who had the *MYD88^L265P^* mutation (TN, *n* = 37; R/R, *n* = 164) [44] and assessed response to zanubrutinib in *MYD88^WT^* patients (TN, *n* = 5; R/R, *n* = 23) [45]. No patients achieved a complete response (CR). In patients with the *MYD88^L265P^* mutation, 29 (28%) who received zanubrutinib and 19 (19%) who received ibrutinib achieved a very good partial response (VGPR); however, these results were not significantly different. Major response rates (MRRs) were 77% and 78%, respectively [44]. The most common AEs (reported in >20% of patients in any treatment arm) were diarrhea (ibrutinib, 32%; zanubrutinib, 21%), upper respiratory tract infection (ibrutinib, 29%; zanubrutinib, 24%), contusion (ibrutinib, 24%; zanubrutinib, 13%), muscle spasm (ibrutinib, 24%; zanubrutinib, 10%), and neutropenia (ibrutinib, 13%; zanubrutinib, 29%). At follow-up (median, 18 months) in patients with *MYD88^WT^*, 7 patients (27%) achieved a VGPR, and 50% had a major response; the estimated PFS and OS rates at 18 months were 68% and 88%, respectively [45].

### Recent Developments

In a long-term follow-up (median, 43 months) to the ASPEN study, the CR combined with VGPR rate in patients with the *MYD88* mutation who received zanubrutinib (*n* = 102) or ibrutinib (*n* = 99) was 36% and 22%, respectively, and 31% in patients without the mutation who received zanubrutinib (*n* = 28) [46]. Similar safety outcomes were observed between patients with and without the *MYD88* mutation who received zanubrutinib. Combined CR and VGPR rates were lower in patients with both the *MYD88* and *CXCR4* mutations than in those with the *MYD88* mutation and wild-type *CXCR4* treated with zanubrutinib (28% and 45%, respectively) or ibrutinib (5% and 21%, respectively), with the rates in both groups being higher for patients treated with zanubrutinib. 

A phase 2 trial of zanubrutinib in Chinese patients with R/R WM (*n* = 44) had efficacy findings similar to those in the initial ASPEN analysis [47]. The MRR in patients with *MYD88^L265P^* and *MYD88^WT^* WM were 73% and 50%, respectively, at the median follow-up time of 33 months. Frequent TEAEs that were grade ≥ 3 included decreased neutrophil count (31.8%), decreased platelet count (20.5%), and pneumonia (20.5%), and no cases of atrial fibrillation/flutter occurred. Together, these data suggest that zanubrutinib can induce durable responses in Chinese patients with R/R WM and that it has an acceptable tolerability profile.

A matching-adjusted indirect comparison of zanubrutinib and rituximab-based chemoimmunotherapy from 2 single-arm studies showed that zanubrutinib resulted in better patient outcomes than either bendamustine-rituximab (BR) (zanubrutinib post-matching, *n* = 50; BR, *n* = 71) or dexamethasone-rituximab-cyclophosphamide (DRC) (zanubrutinib post-matching, *n* = 53; DRC, *n* = 72) [48]. Zanubrutinib resulted in longer PFS and OS compared with both BR (post-matching hazard ratio [95% CI], 0.37 [0.15–0.91] and 0.29 [0.10–0.85], respectively) and dexamethasone-rituximab-cyclophosphamide (post-matching hazard ratio [95% CI], 0.35 [0.14–0.86] and 0.47 [0.14–1.62], respectively), although treatment with zanubrutinib was also associated with a higher incidence of neutropenia post matching (14.3%) compared with dexamethasone-rituximab-cyclophosphamide (9.7%; post-matching risk ratio [95% CI], 1.47 [0.58–3.74]). A lower incidence of neutropenia (17.5%) and pneumonia (1.5%) also occurred with zanubrutinib than BR (35% and 6%; risk ratio [95% CI], 0.50 [0.27–0.91] and 0.26 [0.03–2.28], respectively). These data suggest that zanubrutinib may improve treatment efficacy over traditional chemoimmunotherapy approaches.

Bing-Neel syndrome is a rare complication of WM seen in approximately 1% of patients that occurs when lymphoplasmacytic lymphoma cells enter the central nervous system, causing neurological symptoms [10]. There is no standardized treatment for Bing-Neel syndrome, and therapeutic options are limited to agents that can penetrate the blood–brain barrier. The efficacy of ibrutinib in Bing-Neel syndrome has been reported in a handful of cases [49,50,51,52] and a retrospective multicenter study of 28 patients, which reported improvement in symptomatic and radiologic results in 85% and 60% of patients, respectively, with a 5-year Bing-Neel syndrome survival rate of 86% (95% CI, 63–95%) [53]. One case report has described zanubrutinib use in Bing-Neel syndrome. A 75-year-old woman who developed Bing-Neel syndrome experienced small symptomatic improvements after 12 cycles of high-dose methotrexate, but no improvement was observed on magnetic resonance imaging scans [54]. Treatment with zanubrutinib improved her symptoms, and magnetic resonance imaging scans showed complete resolution of contrast-enhancing lesions in the cervical and thoracic cord and reduced contrast in the intradural lumbar nerve roots. After 15 months of zanubrutinib treatment, the patient had maintained a VGPR. More research on the efficacy of zanubrutinib in Bing-Neel syndrome is warranted.

## 4. Health Economics and Outcomes Research

### 4.1. Early Estimates of Costs for Treating WM

Real-world treatment patterns, adherence, and economic outcomes in patients with WM in the US who have commercial insurance were evaluated in a retrospective observational study using the IBM MarketScan^®^ commercial claims and Medicare supplement database [55]. Data were obtained from patients with WM who were treated with rituximab monotherapy, chemotherapy-based regimens (alone or in combination), proteasome inhibitor-based regimens (alone or in combination with rituximab), ibrutinib (alone or in combination with rituximab), and other regimens between 2014 and 2019, prior to FDA approval of zanubrutinib. Costs for first-line therapy (*n* = 453) were substantial at $19,185 per-patient per-month and increased by line of therapy to $40,452 per-patient per-month for third-line therapy (second-line, *n* = 143; third-line, *n* = 24). This real-world evidence suggests that commercially insured patients in the US have experienced economic burden associated with treatments for WM.

WM has also been tied to productivity losses and increased indirect costs for patients (*n* = 394) and their caregivers (*n* = 190) [56]. Absentee claims, short-term disability, and long-term disability were reported in 82%, 17%, and 3% of patients with indirect costs of $2056, $1177, and $662 per-patient per-month, respectively. Caregivers of patients with WM also had high absentee claims (75%) with indirect per-patient per-month costs of $185. Although short-term disability was lower in caregivers (8%), the per-patient per-month cost was higher ($324). Treatment options that offer timely disease control with an improved side effect profile could prevent or decrease hospitalizations and reduce the economic impact on patients and their caregivers [56,57].

### 4.2. Zanubrutinib: A Cost-Effective Treatment for WM

Models based on data from the ASPEN trial comparing the costs of WM treatment in the US using zanubrutinib or ibrutinib support the idea that zanubrutinib is the more cost-effective option. A partitioned survival model was used to estimate life years, quality-adjusted life years, and costs for patients with WM treated with ibrutinib or zanubrutinib over a 30-year lifetime horizon [58]. Patients who received zanubrutinib experienced a gain of 0.94 life years and 0.84 quality-adjusted life years, with an additional cost of $11,132. The additional cost was driven by patients experiencing a longer time to treatment failure and thus remaining on zanubrutinib longer. An Excel-based model was used to estimate the cost per response for patients with WM treated with ibrutinib or zanubrutinib for 1 year [59]. Total direct medical costs per modeled patient were lower with zanubrutinib than ibrutinib ($152,348 vs. $167,924, respectively). Patients who received zanubrutinib also had a lower cost per response ($544,100 vs. $883,808 with zanubrutinib vs. ibrutinib), suggesting that zanubrutinib is a more cost-effective treatment option than ibrutinib. The lower cost per response was largely driven by drug cost. Studies to compare the cost effectiveness of zanubrutinib with other therapies for WM have not been performed but could be informative for treatment decisions. 

## 5. Future Directions

### 5.1. Zanubrutinib Treatment Combinations in WM

In addition to monotherapy, zanubrutinib has been shown to be effective in other B-cell malignancies when used in combination with additional agents. Combination regimens may also prove to be effective in WM. A preclinical study showed favorable results when zanubrutinib was used in combination with bortezomib for MCL when cells exhibited high expression levels of BTK [60]. Low doses at 40% of the predetermined half-maximal inhibitory concentration for each drug worked synergistically in 3 different MCL cell lines (Jeko-1, Rec-1, and Z138) to produce strong anticancer effects by increasing cell cycle arrest, regulating apoptosis-related proteins, and inhibiting the nuclear factor κB pathway. Additionally, tests showed that zanubrutinib may have a better safety profile in clinical use than ibrutinib because zanubrutinib did not share the off-target effect of inhibiting inducible T-cell kinase in T cells.

Zanubrutinib in combination with obinutuzumab, an anti-CD20 monoclonal antibody that can recognize the CD20 epitope on B cells [61], was used in a phase 1 study to treat patients with CLL/SLL [62]. The ORR in patients with TN and R/R CLL/SLL was 100% (*n* = 20) and 92% (*n* = 23), respectively. The most common AEs included upper respiratory tract infection (51%) and neutropenia (44%); neutropenia was also the most common grade 3/4 AE (31%). Zanubrutinib in combination with obinutuzumab was also assessed in comparison to obinutuzumab monotherapy in patients with R/R follicular lymphoma in the ROSEWOOD study (*n* = 217) [63]. At follow-up (median, 12.5 months), the ORR and CR rate was 68.3% and 37.2%, respectively, with combination therapy and 45.8% and 19.4%, respectively, with obinutuzumab monotherapy. Overall, the treatment combination has been effective and well tolerated and is worth further study in CLL as well as WM.

The combination of zanubrutinib and venetoclax, a B-cell lymphoma 2 antagonist, was studied in an arm of the phase 3 SEQUOIA trial to treat patients with TN CLL/SLL who had the del(17p) mutation [64]. With a median follow-up of 7.9 months, AEs were reported in 29 patients (83%), with serious AEs reported in 4 (11%). Common AEs included diarrhea and neutropenia (5 patients each) and fatigue, nausea, and petechiae (4 patients each); the most frequently reported grade ≥ 3 AEs were neutropenia (*n* = 4) and diarrhea (*n* = 2). In the 31 patients who reached the 3-month efficacy assessment after starting zanubrutinib treatment, the ORR was 97%. These early results show that zanubrutinib in combination with venetoclax may be effective and well tolerated in this high-risk population.

A case study of a 53-year-old man with primary central nervous system lymphoma also suggests that zanubrutinib can be used in combination with chemotherapy [65]. After receiving treatment with a high-dose methotrexate-based regimen for 2 courses, the patient experienced disease progression. Zanubrutinib was added to the high-dose methotrexate and rituximab regimen; with 2 cycles of treatment, symptoms gradually improved, and magnetic resonance imaging scans showed a reduction in cranial masses. After 3 courses of chemotherapy plus zanubrutinib, the patient achieved complete remission.

While these reports are encouraging, not all zanubrutinib combinations have had positive effects on patients. Two patients with relapsed WM participated in a phase 1b dose-escalation study exploring the combination of zanubrutinib and tislelizumab, a novel programmed cell death 1 protein antibody [66]. Both patients experienced acute hemolytic transfusion reactions followed by direct antiglobulin test–negative hemolytic anemia and reticulocytopenia. The authors speculated that tislelizumab caused autoimmune complications and that erythroid precursors may have been the target of the immune attack. At points during treatment, zanubrutinib was withheld, but reinitiation helped control the patients’ lymphoma, which the authors hypothesized contributed to resolution of hemolysis. Since WM is associated with immune dysregulation and autoimmune hemolytic anemia, the authors warned that therapeutic agents harnessing the immune system, like tislelizumab, should be used with caution in patients with WM.

Currently, there are 4 trials in progress, including 1 that focuses on combination therapy, involving zanubrutinib in patients with WM (Table 1). Given the promising results from the few clinical trials and case studies using zanubrutinib in combination with other agents, more research should be conducted to assess its efficacy in combination with other drugs. Combination therapies using ibrutinib may provide good examples of combination therapies to test.

### 5.2. Looking to Ibrutinib and Acalabrutinib Combinations for Inspiration

Given that zanubrutinib, ibrutinib, and acalabrutinib are all covalent BTK inhibitors, with zanubrutinib designed to have improved oral absorption and better target occupancy [34], it is logical to look to effective ibrutinib and acalabrutinib treatment combinations used in WM as inspiration for future combinations with zanubrutinib, but with the added advantage of an improved safety profile. For example, the promising results reported with zanubrutinib in combination with venetoclax or obinutuzumab are not surprising, given similar findings with ibrutinib in combination with venetoclax and obinutuzumab [67]. Patients with both TN and R/R CLL achieved 2-month ORRs of 84% and 88%, respectively, with a tolerable safety profile. Currently, there are 17 active clinical trials using ibrutinib or acalabrutinib in combination with other agents to treat WM (Table 2).

The combination of ibrutinib plus rituximab is one of the preferred treatment regimens for patients with TN or R/R WM, based on improved PFS observed for the combination compared with placebo-rituximab regardless of patient characteristics or mutation status [68]. However, a formal comparison between ibrutinib plus rituximab and ibrutinib monotherapy has not been made. Therefore, the benefit of the addition of rituximab to ibrutinib monotherapy is unclear in WM. Interestingly, studies comparing ibrutinib monotherapy to ibrutinib plus rituximab in patients with CLL have shown no differences in efficacy with combination therapy [69,70]. However, there was a trend for differences in complete remission in patients with TN CLL (20% vs. 50%, respectively) and those with del(17p) and/or *TP53* mutations (22% vs. 33%, respectively) [69].

Ibrutinib in combination with chemoimmunotherapy also has had promising results. The phase 3 HELIOS trial assessed ibrutinib (*n* = 289) or placebo (*n* = 289) in combination with BR, followed by ibrutinib or placebo monotherapy in patients with R/R CLL [71]. PFS rates at 36 months were 68.0% and 13.9%, respectively, demonstrating improved survival outcomes when ibrutinib was used with BR. However, the response to ibrutinib in patients with TN *MYD88^L265P^* WM (*n* = 139) was similar to that observed with BR (*n* = 208; ORR, 94% each) with deeper responses for BR (MRR, 83% vs. 92%; CR, 2% vs. 20%; ≥VGPR, 33% vs. 50) [72]. In a phase 1b study that assessed ibrutinib in combination with BR (*n* = 30) or fludarabine, cyclophosphamide, and rituximab (*n* = 3) to treat R/R CLL, the results support the use of ibrutinib in combination with BR, as the 12- and 36-month PFS rates were 86.3% and 70.3%, respectively, with an ORR of 93.3% [73]. 

Ibrutinib has been used in combination with ulocuplumab, a *CXCR4* antagonist, in a phase 1 study to treat patients with TN or R/R WM who had the *CXCR4* mutation [74]. The ORR and MRR were both 100% (*n* = 12), the VGPR rate was 33% (*n* = 4), and the estimated 2-year PFS rate was 90% (95% CI, 47–99%). Treatment, even at the highest dose of ulocuplumab, was well tolerated and did not increase the rate of AEs. Unfortunately, studies with ulocuplumab were terminated by its sponsors due to poor responses in other diseases; since positive responses were observed with this *CXCR4* antagonist, future research on similar drugs could be considered for patients with WM.

### 5.3. Chimeric Antigen Receptor (CAR) T-Cell Therapy

CAR T-cell therapy has shown good tolerability in patients with WM. In a CAR T-cell dose-escalation study that included 5 patients with R/R WM, no dose-limiting toxicities were observed, and 1 patient with WM experienced a VGPR [75]. In a case study, a 71-year-old man with R/R WM who received CAR T-cell therapy achieved a CR 1 month after treatment that was sustained at the 12-month follow-up visit [76].

Larger studies of CAR T-cell therapy in combination with ibrutinib have shown promising results in patients with R/R CLL/SLL. In 1 cohort of the TRANSCEND CLL 004 phase 1/2 study, 19 patients with R/R CLL/SLL previously treated with ibrutinib received ibrutinib in combination with lisocabtagene maraleucel [77]. Patients started or continued ibrutinib prior to CAR T-cell treatment and continued it for at least 90 days post infusion. Dose-limiting toxicities were not observed in patients at either of 2 dose levels. At the 1-month follow-up, 18 patients experienced an overall response of 95% (100% at the high dose level and 75% at the lower dose level), 9 of which had a CR/CR with an incomplete blood count recovery; at the 3-month follow-up, 15 patients maintained their response. Minimal residual disease was undetectable by flow cytometry in the blood of 17 patients and by next-generation sequencing in the bone marrow of 15 patients.

In a different phase 1/2 study, patients with R/R CLL were given CD19 CAR T-cell therapy with (*n* = 19) or without (*n* = 19) ibrutinib [78]. Comparable ORRs were observed between patients who received ibrutinib and those who did not (83% vs. 56%, respectively; *p* = 0.15). The 1-year OS probability for combination therapy was 64% (95% CI, 42–98%) compared with 61% (95% CI, 42–88%) for CAR T-cell therapy alone (*p* = 0.80). The 1-year PFS probability with combination therapy was comparable to CAR T-cell therapy alone (38% [95% CI, 19–78%] vs. 50% [95%, 31–79%]; *p* = 0.91). These findings suggest that the combination of ibrutinib and CAR T-cell therapy may be beneficial for patients with R/R CLL. As described above, combination approaches involving ibrutinib are worth exploring with zanubrutinib.

### 5.4. Resistance to Covalent BTK Inhibitors

As zanubrutinib use increases, more patients with WM will experience resistance to treatment, as has occurred with ibrutinib [79]. Resistance to ibrutinib has been associated with mutations in *BTK*, particularly *BTK^Cys481^* [79,80], a mutation in the binding site for ibrutinib [81] and zanubrutinib [34]. When combined with gatekeeper mutations, resistance to irreversible BTK inhibitors increases [82]. Since BTK is tied to multiple signaling pathways, resistance can lead to different effects, including increased microRNA expression with specific increases in 14q32 cluster microRNAs, and decreased *pten* mRNA expression [83]. Targeting molecules in pathways associated with BTK, such as 14q32 cluster microRNAs, could be beneficial [83]. Combination approaches should be explored to identify effective treatment strategies that allow patients to use BTK inhibitors while reducing the risk of resistance in patients with WM and potentially offering a limited duration therapy. Newer treatment options, such as non-covalent BTK inhibitors, which display more variable resistance patterns to mutations [82], can also be beneficial for circumventing issues with resistance to covalent BTK inhibitors. 

### 5.5. Newer BTK Therapies for WM

BTK inhibitors have a positive effect on patients with WM, and reversible BTK inhibitors, such as nemtabrutinib and pirtobrutinib, are now being explored for use in B-cell malignancies. Preclinical evidence shows that nemtabrutinib inhibits CLL cell survival and suppresses activation of mutations that facilitate resistance to ibrutinib [84]. Pirtobrutinib was evaluated in the phase 1/2 BRUIN clinical trial in 323 patients with R/R B-cell malignancies, including 26 patients with WM [85]. In 139 efficacy-evaluable patients with CLL/SLL, the ORR was 63% (95% CI, 55–71%); in 121 efficacy-evaluable patients previously treated with BTK inhibitors, the ORR was 62% (95% CI, 53–71%). 

The identification of the dual BTK and hematopoietic cell kinase inhibitor KIN-8194 also has great potential for future treatment of WM [86]. In vitro research showed potent and selective death of cells with mutations in *MYD88*, including cells that expressed *BTK^Cys481Ser^*, which is associated with ibrutinib resistance. Antitumor activity was more potent than with ibrutinib, although no effect was observed in *MYD88*-mutated OCI-Ly3 cells with the caspase recruitment domain family member 11 (*CARD11*) mutation. In rodent models, KIN-8194 demonstrated excellent bioavailability and PK properties with good tolerance. After treatment was ceased, mice who received KIN-8194 had undetectable or stable tumors, while mice treated with ibrutinib experienced tumor growth.

### 5.6. Important Considerations for BTK Inhibitor Therapy

Recent research suggests that BTK inhibitors can also support COVID-19 recovery in patients with [87] and without B-cell malignancies [88], including those who have WM [89,90]. However, concurrent use of BTK inhibitors in patients who received the COVID-19 vaccine was associated with a markedly reduced response to vaccination [18]. Proper planning around treatment and vaccination is warranted.

## 6. Conclusions

Great progress has been made in the treatment of patients with WM, and novel agents are still being produced to help curb the devastating effects of this disease. With demonstrated improvements in the safety profile over ibrutinib, zanubrutinib may provide patients with flexible dosing, a better tolerated treatment option, and beneficial effects of BTK inhibition. Zanubrutinib may offer the potential to reduce the negative economic impact experienced during treatment of WM when compared with ibrutinib. Combination therapies should be the focus of future research to help prevent development of resistance using therapies of finite duration. Emerging therapies may change the treatment landscape for WM, but BTK inhibition currently remains a cornerstone of therapy.

## Figures and Tables

**Table 1:** Ongoing clinical trials of zanubrutinib in patients with Waldenström macroglobulinemia.

ClinicalTrials.gov ID	Title	Agents	Phase	Eligibility
NCT04463953	Zanubrutinib, Ixazomib and Dexamethasone in Patients with Treatment Naive Waldenstrom’s Macroglobulinemia	Zanubrutinib, ixazomib, and dexamethasone	2	TN
NCT04116437	Zanubrutinib (BGB-3111) in Participants With Previously Treated B-Cell Lymphoma Intolerant of Prior Bruton Tyrosine Kinase Inhibitor (BTKi) Treatment	Zanubrutinib	2	Ibrutinib or acalabrutinib intolerant
NCT04172246	Study of Zanubrutinib in Japanese Participants With B-Cell Malignancies	Zanubrutinib	1/2	R/R
NCT03053440	A Study Comparing BGB-3111 and Ibrutinib in Participants WithWaldenström’s Macroglobulinemia (WM) (ASPEN)	Zanubrutinib and ibrutinib	3	No prior BTK inhibitor exposure

BTK, Bruton tyrosine kinase; R/R, relapsed/refractory; TN, treatment naive.

**Table 2:** Ongoing clinical trials using ibrutinib or acalabrutinib combination therapy in patients with Waldenström macroglobulinemia.

ClinicalTrials.gov ID	Title	BTKi Combination Agents	Phase	Eligibility
NCT03620903	Efficacy of First Line B-RI for Treatment Naive Waldenström’s Macroglobulinemia	Bortezomib, rituximab, and ibrutinib	2	TN
NCT04062448	A Study of Ibrutinib in Combination with Rituximab, in Japanese Participants with Waldenstrom’s Macroglobulinemia (WM)	Ibrutinib and rituximab	2	Japanese
NCT03225716	A Study of Ulocuplumab And Ibrutinib in Symptomatic Patients with Mutated *CXCR4* Waldenstrom’s Macroglobulinemia	Ulocuplumab and ibrutinib	1/2	*MYD88* and *CXCR4* mutated disease
NCT04263480	Efficacy and Safety of Carfilzomib in Combination with Ibrutinib vs. Ibrutinib in Waldenström’s Macroglobulinemia (CZAR-1)	Carfilzomib and ibrutinib	3	No prior exposure to a BTKi or carfilzomib
NCT04274738	A Study of Mavorixafor in Combination with Ibrutinib in Participants with Waldenstrom’s Macroglobulinemia (WM) Whose Tumors Express Mutations in *MYD88* and *CXCR4*	Ibrutinib and mavorixafor	1	*MYD88^L265P^* and *CXCR4^WHIM^* mutations
NCT04061512	Rituximab and Ibrutinib (RI) Versus Dexamethasone, Rituximab and Cyclophosphamide (DRC) as Initial Therapy for Waldenström’s Macroglobulinaemia (RAINBOW)	Ibrutinib and rituximab	2/3	TN
NCT04273139	Ibrutinib + Venetoclax in Untreated WM	Ibrutinib and venetoclax	2	*MYD88* mutation
NCT04260217	APG-2575 Single Agent or in Combination with Ibrutinib or Rituximab in Patients with Waldenström Macroglobulinemia (MAPLE-1)	Ibrutinib and lisaftoclax (APG-2575)	1b/2	TN
NCT03679624	Daratumumab Plus Ibrutinib in Patients with Waldenström’s Macroglobulinemia	Daratumumab and ibrutinib	2	Ibrutinib-naive or current treatment on ibrutinib with a plateaued response
NCT02332980	Pembrolizumab Alone or with Idelalisib or Ibrutinib in Treating Patients with Relapsed or Refractory Chronic Lymphocytic Leukemia or Other Low-Grade B-Cell Non-Hodgkin Lymphomas	Ibrutinib and pembrolizumab	2	R/R
NCT01955499	Lenalidomide and Ibrutinib in Treating Patients with Relapsed or Refractory B-Cell Non-Hodgkin Lymphoma	Ibrutinib and lenalidomide	1	R/R
NCT03479268	Pevonedistat and Ibrutinib in Treating Participants with Relapsed or Refractory CLL or Non-Hodgkin Lymphoma	Pevonedistat and ibrutinib	1	R/R
NCT01479842	Rituxan/Bendamustine/PCI-32765 in Relapsed DLBCL, MCL, or Indolent Non-Hodgkin’s Lymphoma	Ibrutinib (PCI-32765), rituximab, and bendamustine	1	R/R
NCT04624906	Bendamustine, Rituximab and Acalabrutinib in Waldenstrom’s Macroglobulinemia (BRAWM)	Acalabrutinib, bendamustine, and rituximab	2	TN
NCT05065554	ACALA-R In Anti-MAG Neuropathy Mediated Neuropathy	Acalabrutinib and rituximab	2	Sensory neuropathy
NCT02362035	ACP-196 (Acalabrutinib) in Combination WITH Pembrolizumab, for Treatment of Hematologic Malignancies (KEYNOTE145)	Acalabrutinib and pembrolizumab	1b/2	Hematological malignancy
NCT04883437	Acalabrutinib and Obinutuzumab for the Treatment of Previously Untreated Follicular Lymphoma or Other Indolent Non-Hodgkin Lymphomas	Acalabrutinib and obinutuzumab	2	TN

BTKi, Bruton tyrosine kinase inhibitor; R/R, relapsed/refractory; TN, treatment-naive.

## Data Availability

Data sharing is not applicable to this article as no data sets were generated or analyzed for this review.
